# Preparation and Characterization of Nanocellulose/Chitosan Aerogel Scaffolds Using Chemical-Free Approach

**DOI:** 10.3390/gels7040246

**Published:** 2021-12-02

**Authors:** Samsul Rizal, Esam Bashir Yahya, H. P. S. Abdul Khalil, C. K. Abdullah, Marwan Marwan, Ikramullah Ikramullah, Umar Muksin

**Affiliations:** 1 Department of Mechanical Engineering, Universitas Syiah Kuala, Banda Aceh 23111, Indonesia; Ikramullah@mhs.unsyiah.ac.id; 2 School of Industrial Technology, Universiti Sains Malaysia, Penang 11800, Malaysia; ck_abdullah@usm.my; 3 Department of Chemical Engineering, Universitas Syiah Kuala, Banda Aceh 23111, Indonesia; marwan@unsyiah.ac.id; 4 Department of Physics, Universitas Syiah Kuala, Banda Aceh 23111, Indonesia; muksin.umar@unsyiah.ac.id

**Keywords:** aerogel scaffold, cellulose nanofibers, chitosan, green materials, medical applications

## Abstract

Biopolymer-based aerogels are open three-dimensional porous materials that are characterized by outstanding properties, such as a low density, high porosity and high surface area, in addition to their biocompatibility and non-cytotoxicity. Here we fabricated pure and binary blended aerogels from cellulose nanofibers (CNFs) and chitosan (CS), using a chemical-free approach that consists of high-pressure homogenization and freeze-drying. The prepared aerogels showed a different porosity and density, depending on the material and mixing ratio. The porosity and density of the aerogels ranged from 99.1 to 90.8% and from 0.0081 to 0.141 g/cm^3^, respectively. Pure CNFs aerogel had the highest porosity and lightest density, but it showed poor mechanical properties and a high water absorption capacity. Mixing CS with CNFs significantly enhance the mechanical properties and reduce its water uptake. The two investigated ratios of aerogel blends had superior mechanical and thermal properties over the single-material aerogels, in addition to reduced water uptake and 2-log antibacterial activity. This green fabrication and chemical-free approach could have great potential in the preparation of biopolymeric scaffolds for different biomedical applications, such as tissue-engineering scaffolds.

## 1. Introduction

Aerogels are porous materials of interconnected nanostructures made from numerous materials by replacing the liquid in their suspension/gels with gas [1]. Aerogels exhibit unusual properties, such as high porosity and surface area, low density and low heat conductivity. The past two decades have witnessed the preparation of aerogels from different organic and inorganic materials, including silica, alumina, tin oxide, chromia and carbon aerogels, with silica remaining the most widely used one. Biopolymers are naturally occurring polymers produced by living cells from animal, plants or microorganisms [2]. They are preferably used in biomedical applications due to their biocompatibility and non-cytotoxicity [3,4]. Biopolymers-based aerogels are a type of material that is characterized by a high surface area, low density and light weight [5,6]. Such aerogels, cellulose- and chitosan-based, in particular, have received a remarkable boost in both academic researches and industrial biomedical applications [6,7]. Owing to their unique properties, such as easy scale-up, ecofriendly, non-cytotoxicity and biocompatibility, biopolymers-based aerogels have been used in drug delivery [5], wound healing and dressing [8], biosensing [9] and tissue scaffolding [10].

The mechanical properties and behavior of biopolymeric aerogels are essential for many applications, such as tissue-engineering scaffolds [11]. Pure-cellulose and pure-chitosan aerogels are intrinsically hydrophilic, as they highly uptake water and eventually undergo a partial irreversible collapse [12]. Different modifications have been applied to these aerogels, such as using chemical crosslinkers and changing the fabrication technique [13,14]. Reinforcement of aerogel with materials such as silica or graphene oxide was found to significantly reduce the water uptake of cellulose aerogel, increase its hydrophobicity and enhance the mechanical properties. Ge et al. [15] used graphene oxide to enhance the mechanical strength of cellulose aerogel. Others used ester crosslinking of CNFs aerogel by gas–solid fluorinating reaction, with or without organic solvent assistance [16]. However, as non-biocompatible materials, introducing silica or other inorganic substances could reduce the biocompatibility of the resulted aerogels, thus limiting their applications in biomedical fields.

A moderate-water-absorption characteristic is required in tissue scaffolding; the scaffold should not be hydrophobic and not too hydrophilic that it could collapse upon loading the cells. Moreover, the surfaces of the scaffolds should allow the attachment of cells; thus, its highly desirable to use biocompatible and non-cytotoxic material in scaffold fabrication. A nanocellulose and collagen aerogel was found to have strong water absorption of more than 4000%, which is even higher than the pure CNFs aerogel [17]. CS has been widely used in biomedical applications, due to its antibacterial activity, in addition to the other properties of biopolymers. Mousumi et al. [18] fabricated an oxidized cellulose/chitosan aerogel by using a lyophilization process and reported a poor swelling ratio, in addition to weak mechanical strength. In a recent work, Zhang et al. [19] used chitosan to enhance the mechanical properties and water uptake of nanocellulose and reported the ability of their aerogel in oil/seawater mixtures’ separation. Directional freeze-casting and a chemical-crosslink process were used in a different study in the preparation of a CNF/CS aerogel with anisotropic thermal management properties [20]. Although the homogenization and freeze-drying routs of fabrication have been used in previous publications, they have not indicated the exact role of CS in enhancing the morphological, mechanical, thermal and in vitro water absorption properties of pure CNFs. In the present work, we aimed to enhance the properties of a nanocellulose aerogel by introducing chitosan in different ratios and evaluated the composite aerogels compared with the pure ones, using a chemical-free approach. This study provides evidence that CNFs/CS aerogels have superior properties when compared to pure-CNFs and pure-CS aerogels.

## 2. Results and Discussion

**Figure 1:**
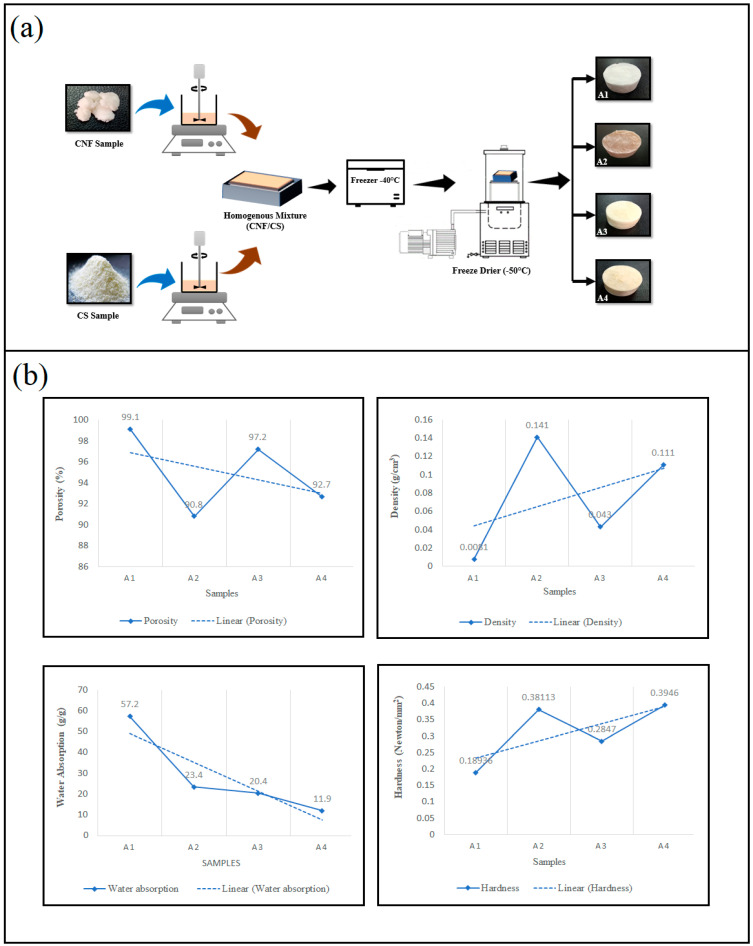
Properties of CNFs and CS aerogels: (**a**) the overall preparation procedure and (**b**) the morphological analysis of the four samples (density, porosity, water absorption and hardness); A1 = pure CNFs aerogel, A2 = pure CS aerogel, A3 = 60/40 CNFs/CS aerogel and A4 = 40/60 CNFs/CS aerogel.

Aerogels’ processing in this work started with high-pressure homogenization of the polymeric powder in distilled water, followed by direct freezing for 24 h and freeze-drying (Figure 1a), which differ from the conventional approaches that form hydrogel prior to the drying phase.

### 2.1. Morphological Analysis

Pure CNFs aerogel (A1) had the lowest density value of 0.0081 g/cm^2^ when compared with pure chitosan sample (A2) and the two blends (A3 and A4). However, the results in Figure 1b show that, with the increase of chitosan, the density increases, as well as the mechanical strength. A1 had an ultralight weight and density, and, thus, it had the highest porosity (99.1) when compared with the lowest porosity 90.8 (A2). Owing to its nanosize and high surface area, pure CNFs aerogel, as expected, show the highest porosity; the strength was minimum due to the weak adhesion of CNFs molecules with each other [21]. Interestingly, the hardness of A4 samples, which are composed of 60:40% CS–CNFs, had the highest hardness value of 0.3946 N/mm^2^, which is even higher than the pure CS itself. This improvement can be explained by better interconnection of nanosized CNFs molecules and CS, which crosslinked together and enhanced the interactions among building blocks [22]. Gupta et al. [23] reported the fabrication of CNFs aerogel with 99.4% porosity and the same density of the one obtained in our study. The density of the aerogel is inversely in proportion to its porosity; the higher porosity, the lower the density and poorer the mechanical properties [3]. Cellulose is characterized by high hydrophilicity [24], and, thus, the aerogel containing higher CNFs possessed higher water absorption. It can be observed that aerogels with lower porosity exhibit higher hardness and a lower water absorption level. Introducing chitosan into the aerogels significantly increases their hardness and lowers the hydrophobicity.

**Figure 2:**
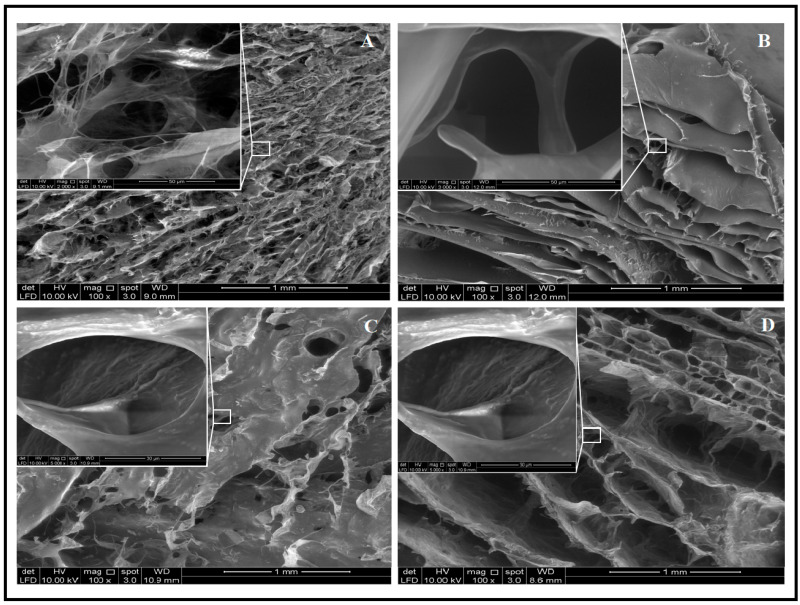
Field-Emission Scanning Electron Microscope (FE-SEM) images of aerogels: (**A**) pure-CNFs aerogel, (**B**) pure-CS aerogel, (**C**) 60–40 CNFs/CS and (**D**) 40–60 CNFs/CS.

A Field-Emission Scanning Electron Microscope analysis of the aerogels is presented in Figure 2. The difference between the pores’ morphology and structure of CNFs and CS can clearly be observed; pure CNFs had smaller pores compared with CS aerogel. However, the surface of the aerogel was smoother with the increase in chitosan, and this could explain the increase in the hardness and hydrophobicity of the aerogels [25].

### 2.2. Surface Functional Groups

**Figure 3:**
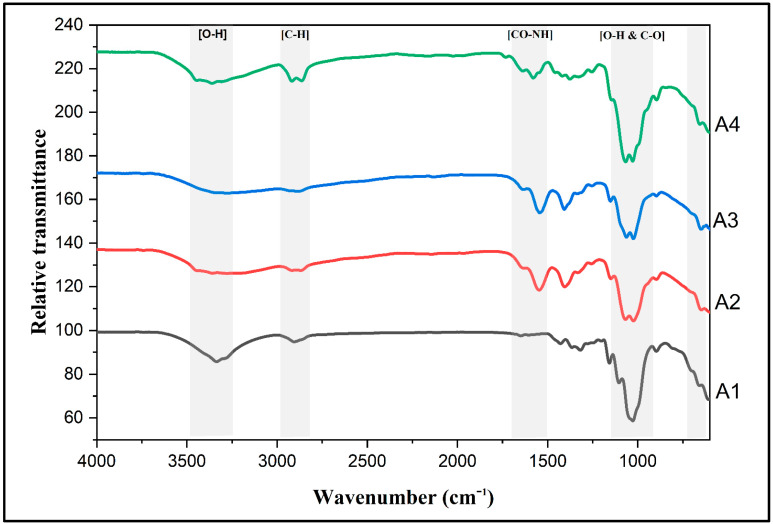
Fourier-transform infrared (FTIR) spectra of prepared aerogel samples.

Figure 3 compares the results of the surface functional groups of the prepared aerogel samples to assess the variations of any possible chemical structure changes. From the FTIR spectra, we see that A1 showed clear sharp peak of –OH vibrations at 3332.9 cm^−1^, caused by intra-molecular hydrogen bonding [26]. After incorporating the CS matrix into the CNFs, the intensity of the –OH peak markedly reduced, thus confirming the hydrogen-bonding formation between CNFs and CS [27]. The peaks at around 2916 and 2856 cm^−1^ correspond to symmetric and asymmetric C–H vibrations, respectively [28]. These peaks were presented at a high intensity in sample A4, with a higher chitosan concentration. The same sample showed higher intensity in the peaks at 1070 and 1024, cm^−1^, thus reflecting the bending of O–H and stretching of C–O. Moreover, in higher CNFs-content samples (A1 and A4), the intensity of 1319 cm^−1^ was higher. Comparatively, CS samples showed clear peaks at 1651 and 1546 cm^−1^, reflecting the vibrational mode of the amide II and I groups, respectively [29].

### 2.3. Texture Profile Analysis

The results of the texture profile analysis for all the prepared aerogel samples are presented in Table 1. Pure-CNFs aerogel had the lowest mechanical values among all the samples; the introduction of CS significantly enhanced the mechanical properties of aerogels. However, the A4 sample containing 40/60 CNFs/CS showed the maximum strength of 4023.8 g, compared with the pure-CS sample (A2), which showed only 3886.4 g. This improvement can be explained by both the large specific surface area of CNFs and strong adhesion properties of CS that crosslinked among the CNFs molecules and enhanced the interactions among building blocks [22]. The designed anisotropic structure can also sustain large stress through the densification effect, thus enhancing the mechanical properties upon the addition of CS to the system. Considering the strength of aerogels, we observed that the resilience of pure CS was very high compared with CNFs. However, in this regard, the A4 samples had the best mechanical properties among all the samples; this can be explained by the role of CNFs in enhancing the mechanical properties of materials [30].

### 2.4. Thermal Properties

**Figure 4:**
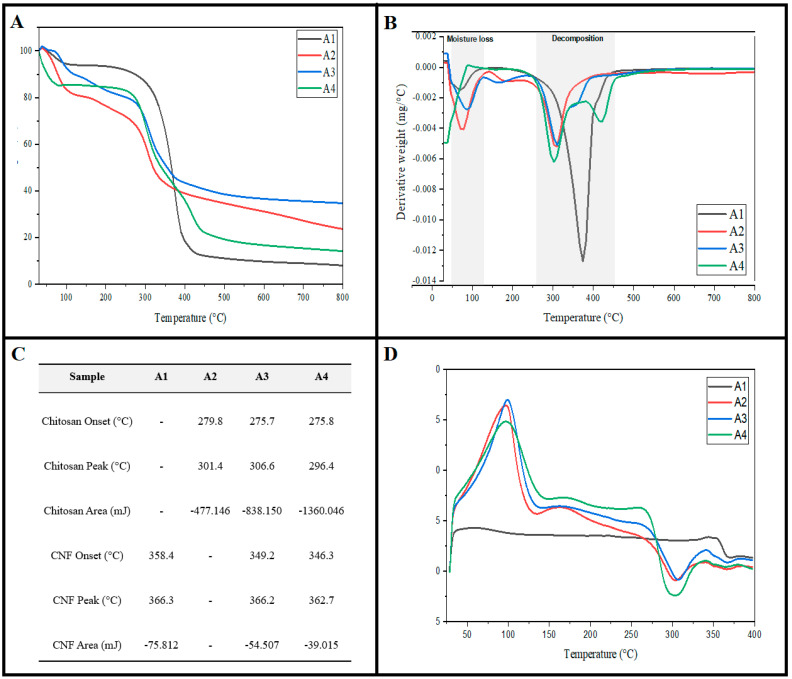
Thermal properties of aerogel samples: (**A**) thermo-gravimetry analysis (TGA) curves, (**B**) derivative thermo-gravimetry analysis (DTG) curves and (**C**,**D**) differential scanning calorimetry (DSC) analysis.

Cellulosic materials are known for their thermal sensitivity, which normally degrades at low-to-moderate temperatures [31]. The addition of CS to CNFs aerogel significantly alters the thermal properties, as shown in Figure 3. Thermogravimetry analysis (TGA) curves (Figure 4a) show that A1 had a T_onset_ of 358.4 °C, compared with A2 (279.8 °C). The mixed aerogels A3 and A4 show two temperature onsets of 275.7 and 349.2 °C for A3 and 275.8 and 346.3 °C for A4. At a low-temperature, i.e., around 100 °C, evaporation of moisture, physisorbed water and volatile compounds occur, leading to a slight weight loss of approximately 10% [32]. Compared with CNFs, CSs tend to have a larger amount of bound water, due to the weakening of the hydrogen bonding during the gelation and dry phases [33]; this explains the flat light in the DSC graph in Figure 4d. The aerogel samples had a decomposition temperature lower than 400 °C, regardless of the mixing ratio of CNFs and CS. The pure CNFs sample showed the highest decomposition temperature, at 375 °C, followed by A3 (315 °C) and, finally, pure CS and A4, with 306 and 303 °C, respectively. The increase in the CNF/CS aerogel’s decomposition temperature was mainly due to the high thermal stability of chitosan and the strong interactions between the CNF and chitosan molecules. Our results were similar to those obtained in the study of Zhang et al. [20]. Interestingly, the addition of CNFs to CS reduced the decomposition temperature even more than the pure CS itself; this could be due to the crosslinking between CNFs and CS. Neto et al. [34] reported that a low degree of chemical crosslinking tends to lower the decomposition temperature, in contrast to the high degree; this explains the difference between A3 and A4 samples.

### 2.5. In Vitro Water Uptake Evaluation and Viable Bacterial Reduction Test

In order to determine the water uptake of our prepared aerogels, pure aerogels (A1 and A2) were dissolved in media, and we were not able to measure their weight after 7 and 14 days. However, A3 and A4 remained intact even after 14 days inside the saline media, and their water uptake is presented in Table 2. The water absorption capacity was higher in the higher-CNFs sample, and way lower in the chitosan one, due to the large number of hydrophilic groups within the nanocellulose and the pore-rich network structure compared with the chitosan, which can significantly help pure CNFs aerogel to absorb more water [35]. The nanocellulose-reinforced 3D interconnected network structure guaranteed the mechanical performance of the CNFs/CS aerogel. Together the inherent hydrophilicity of CS with the rough microstructure of the aerogel, excellent underwater superoleophobicity was developed in both composite aerogels, similar to the one obtained in the previous study of Zhang et al. [19]. The logarithm reduction of aerogel samples was tested against Gram-negative and Gram-positive bacteria (*E. coli* and *S. aureus*, respectively). The samples with pure and a high percentage of chitosan (A2 and A4) showed 2-log reduction of both bacteria; the initial bacterial count (control sample) was 1.34 × 10^8^ and 1.56 × 10^8^ for *E. coli* and *S. aureus*, respectively, which reduced by two folds of reduction. However, the A1 sample did not show any reduction, as it was free of CS, which is known for antibacterial activity due to the interaction that occurs between CS positive charges and the microbial membrane negative charges [36].

## 3. Conclusions

Pure and blended cellulose nanofiber and chitosan aerogels were successfully synthesized by using a chemical-free approach based on high-pressure homogenization and freeze-drying. Pure CNFs aerogel had the highest porosity and lowest density, but it showed the poorest mechanical properties; thus, it could not remain in vitro intact for more than one day. Introducing chitosan into the CNFs significantly enhanced the mechanical properties of the aerogel, reduced its water uptake and made the aerogel remain in vitro intact. The interaction between CNFs and CS showed good porosity, the best in vitro water uptake and marked antibacterial activity. Chemical-free CNFs/Cs aerogel have great potential in biomedical applications, such tissue-engineering scaffolds.

## 4. Materials and Methods

### 4.1. Materials

CNFs was isolated from Kenaf paste fibers and characterized by using the method reported by Atiqah et al. [37]. Chitosan was obtained from Tokyo Chemical Industry Co., Ltd., Tokyo, Japan, and acetic acid was procured from Merck, Darmstadt, Germany.

### 4.2. Preparation of Aerogel

The preparation of the aerogels started with homogenizing the CNFs in distilled water (4 wt%) for 6 h, using Ultra-turrax homogenizer (IKA, Staufen, Germany). 50 mL of the homogenized CNFs suspension was poured in a container and frozen for 24 h. The CS was dissolved in 1% acetic acid (5 wt%) and magnetically stirred for 2 h until clear solution was obtained. The same amount of CS was taken, and in a container, it was frozen for 24 h. Two different ratios of CNFs and CS were mixed (60/40 CNFs-CS and 40/60 CNFs-CS) and homogenized for 30 min to ensure the complete mixing of molecules; finally, the same amount of each ration was taken and frozen. The four frozen samples, namely pure CNFs (A1), pure CS (A2), 60/40 CNFs-CS (A3) and 40/60 CNFs-CS (A4), were directly placed in a freeze-dryer for 48 h and then characterized.

### 4.3. Density, Porosity and Water Absorption Capacity

The density of aerogels was measured by keeping a constant shape for all the aerogels (1 cm^3^) prepared, and then their mass and volume were measured. The porosity was measured by following the below formula from the calculated density.

$$\mathrm{Porosity}\;\%\;=1-\frac{\mathrm{aerogel}\;\mathrm{density}}{\mathrm{bulk}\;\mathrm{density}}\times 100\;\%$$

The water absorption capacity was determined by immersing 1 cm^3^ of each sample into 20 mL of distilled water and allowing them to saturate for a few minutes. The excess water was then blotted with filter paper, and the saturated aerogel was weighed. Finally, the water absorption capacity was measured as follows:

$$\mathrm{Water}\;\mathrm{absorption}\;\mathrm{capacity}\;=\frac{\mathrm{weight}\;\mathrm{of}\;\mathrm{saturated}\;\mathrm{aerogel}-\mathrm{initial}\;\mathrm{weight}\;}{\mathrm{initial}\;\mathrm{weight}\;}$$

### 4.4. Surface Morphology and Functional Groups

The surface morphology was observed under Field Emission Scanning Electron Microscope (FE-SEM) model Leo Supra, 50 VP, Carl Zeiss, SMT (Carl Zeiss Group, Oberkochen, Germany) with high resolution. Thin layer of each sample was prepared for the microscopic analysis. FT-IR spectroscopy (Thermo Scientific model Nicolet I S10 spectrometer, Thermo Fisher Scientific, Waltham, MA, USA) was used to investigate the functional groups of each aerogel sample, using a Perkin Elmer spectrum 1000 for obtaining the spectrum.

### 4.5. Thermal Properties Analysis

The thermal properties of the aerogel samples were studied by determining the thermo-gravimetric analysis (TGA) and differential scanning calorimetry (DSC). A thermo-gravimetric analyzer (TGA/SDTA 851e, Brand Mettler Toledo, Mettler-Toledo International Inc., Columbus, OH, USA) was used to characterize all TGA curves, while DSC was performed in the DSC unit (Netzsch DSC-200 PC Phox, NETZSCH Holding. Erich NETZSCH GmbH & Co. Holding KG, Selb, Germany). Then 10 mg by weight of each sample was measured, put in a standard cup, placed in the thermogravimetry analyzer with a pre-weighed empty cup as a reference and the samples were heated from 30 to 800 °C. A heating rate of 20 °C/min was used, and the weight loss and derivative weight loss with temperature was obtained under nitrogen.

### 4.6. Mechanical Properties

The mechanical properties of the aerogels were studied by texture profile analysis, using a TA-HDi textile analyzer machine (Stable Micro Systems, Surrey, UK), after cutting the sample into constant length, width and height of 2 × 2 × 1 cm, respectively. The samples were compressed twice to 75% of the original height, and texture profile analyses were determined by two compression cycles. Texture profile parameters measured include hardness, cohesiveness, resilience, gumminess, springiness and chewiness. The analysis was performed with triplicates.

### 4.7. In Vitro Water Uptake Evaluation and Viable Bacterial Reduction Test

The swelling capability of all prepared aerogels was determined after 1, 7 and 14 days, by immersing the 1 cm^3^ of each sample in normal saline solution and inculpated at 37 °C [38]. The weight of the swollen aerogels for each time point was then measured, and the water uptake value was calculated as follows:

(1) $$\mathrm{Water}\;\mathrm{uptake}=\frac{\mathrm{swollen}\;\mathrm{aerogel}-\mathrm{initial}\;\mathrm{weight}\;}{\mathrm{initial}\;\mathrm{weight}}$$

The antibacterial activity of the prepared aerogels was determined by bacterial reduction test of two types of microorganisms, namely *Escherichia coli* and *Staphylococcus aureus*. Viable cell-counting approach was used for determining the bacterial reduction, using sterile normal saline with a bacterial count of 1.34 × 10^8^ and 1.56 × 10^8^ cfu (colony forming unit) for *Escherichia coli* and *Staphylococcus aureus*, respectively. Constant weight of each sample (100 mg) was assigned to the experiment and placed in a tube containing 10 mL of each bacterial suspension and incubated in a shaker at 37 °C for 24 h, and the viable numbers of survival bacteria were observed by using a serial-dilution technique. An untreated tube (aerogel free) was used as the control.

### 4.8. Statistical Analysis

The entire experiment was replicated three times, at different times, in the same place. The data obtained were first calculated by using Microsoft Office Excel 2010 (Microsoft Corporation, Redmond, WA, USA) and ANOVA software for analyzing the data.

## Figures and Tables

**Table 1:** Results of texture profile analysis of prepared aerogel samples (mean ± S.D).

Sample	Hardness (g)	Springiness (mm)	Cohesiveness (%)	Gumminess	Chewiness	Resilience (%)
A1	1931.2 ± 22.3	0.458 ± 0.02	0.408 ± 0.002	787.3 ± 09	360.3 ± 12	0.150 ± 0.007
A2	3886.4 ± 56.2	1.197 ± 0.07	0.754 ± 0.004	1494.8 ± 11	1789.9 ± 08	0.305 ± 0.005
A3	2903.1 ± 13.4	0.728 ± 0.01	0.342 ± 0.002	992.6 ± 07	722.6 ± 10	0.097 ± 0.001
A4	4023.8 ± 17.3	0.812 ± 0.009	0.452 ± 0.01	1818.9 ± 13	1477.1 ± 18	0.141 ± 0.001

**Table 2:** In vitro water uptake and viable bacterial reduction of aerogel samples (Sample A1 and A2 referred to as not applicable (N.A.) as they have collapsed in the water after 7 days).

Sample	0 Day (g/g)	7 Days (g/g)	14 Days (g/g)	Log Reduction of *E. coli*	Log Reduction of *S. aureus*
A1	57.2	N.A	N.A	0-log	0-log
A2	23.4	N.A	N.A	2-log	2-log
A3	20.4	23.6	23.8	1-log	1-log
A4	11.9	19.2	20.4	2-log	2-log

## Data Availability

Not applicable.

## References

[1] Lee J.-H., Park S.-J. (2020). Recent advances in preparations and applications of carbon aerogels: A review. Carbon.

[2] Nuryawan A., Abdullah C., Hazwan C.M., Olaiya N., Yahya E.B., Risnasari I., Masruchin N., Baharudin M., Khalid H., Abdul Khalil H.P.S. (2020). Enhancement of Oil Palm Waste Nanoparticles on the Properties and Characterization of Hybrid Plywood Biocomposites. Polymers.

[3] Abdul Khalil H.P.S., Adnan A., Yahya E.B., Olaiya N., Safrida S., Hossain M., Balakrishnan V., Gopakumar D.A., Abdullah C., Oyekanmi A. (2020). A Review on plant cellulose nanofibre-based aerogels for biomedical applications. Polymers.

[4] Abdul Khalil H.P.S., Jummaat F., Yahya E.B., Olaiya N., Adnan A., Abdat M., NAM N., Halim A.S., Kumar U., Bairwan R. (2020). A review on micro-to nanocellulose biopolymer scaffold forming for tissue engineering applications. Polymers.

[5] Yahya E.B., Jummaat F., Amirul A., Adnan A., Olaiya N., Abdullah C., Rizal S., Mohamad Haafiz M., Abdul Khalil H.P.S. (2020). A review on revolutionary natural biopolymer-based aerogels for antibacterial delivery. Antibiotics.

[6] Zhao S., Malfait W.J., Guerrero-Alburquerque N., Koebel M.M., Nyström G. (2018). Biopolymer aerogels and foams: Chemistry, properties, and applications. Angew. Chem. Int. Ed..

[7] Smirnova I., Gurikov P. (2018). Aerogel production: Current status, research directions, and future opportunities. J. Supercrit. Fluids.

[8] Franco P., Pessolano E., Belvedere R., Petrella A., De Marco I. (2020). Supercritical impregnation of mesoglycan into calcium alginate aerogel for wound healing. J. Supercrit. Fluids.

[9] Li Y., Zhao M., Chen J., Fan S., Liang J., Ding L., Chen S. (2016). Flexible chitosan/carbon nanotubes aerogel, a robust matrix for in-situ growth and non-enzymatic biosensing applications. Sens. Actuators B Chem..

[10] Ghafari R., Jonoobi M., Amirabad L.M., Oksman K., Taheri A.R. (2019). Fabrication and characterization of novel bilayer scaffold from nanocellulose based aerogel for skin tissue engineering applications. Int. J. Biol. Macromol..

[11] Yahya E.B., Amirul A., Abdul Khalil H.P.S., Olaiya N.G., Iqbal M.O., Jummaat F., AK A.S., Adnan A. (2021). Insights into the Role of Biopolymer Aerogel Scaffolds in Tissue Engineering and Regenerative Medicine. Polymers.

[12] Rege A., Ratke L., Külcü İ.D., Gurikov P. (2020). Stiffening of biopolymer aerogel networks upon wetting: A model-based study. J. Non-Cryst. Solids.

[13] Ganesan K., Barowski A., Ratke L., Milow B. (2019). Influence of hierarchical porous structures on the mechanical properties of cellulose aerogels. J. Sol-Gel Sci. Technol..

[14] Vasil’kov A., Rubina M., Naumkin A., Buzin M., Dorovatovskii P., Peters G., Zubavichus Y. (2021). Cellulose-Based Hydrogels and Aerogels Embedded with Silver Nanoparticles: Preparation and Characterization. Gels.

[15] Ge X., Shan Y., Wu L., Mu X., Peng H., Jiang Y. (2018). High-strength and morphology-controlled aerogel based on carboxymethyl cellulose and graphene oxide. Carbohydr. Polym..

[16] Li Y., Liu Y., Liu Y., Lai W., Huang F., Ou A., Qin R., Liu X., Wang X. (2018). Ester crosslinking enhanced hydrophilic cellulose nanofibrils aerogel. ACS Sustain. Chem. Eng..

[17] Lu T., Li Q., Chen W., Yu H. (2014). Composite aerogels based on dialdehyde nanocellulose and collagen for potential applications as wound dressing and tissue engineering scaffold. Compos. Sci. Technol..

[18] Sukul M., Ventura R.D., Bae S.H., Choi H.J., Lee S.Y., Lee B.T. (2017). Plant-derived oxidized nanofibrillar cellulose-chitosan composite as an absorbable hemostat. Mater. Lett..

[19] Zhang H., Li Y., Shi R., Chen L., Fan M. (2018). A robust salt-tolerant superoleophobic chitosan/nanofibrillated cellulose aerogel for highly efficient oil/water separation. Carbohydr. Polym..

[20] Zhang M., Jiang S., Han F., Li M., Wang N., Liu L. (2021). Anisotropic cellulose nanofiber/chitosan aerogel with thermal management and oil absorption properties. Carbohydr. Polym..

[21] Jummaat F., Yahya E.B., Abdul Khalil H.P.S., Adnan A., Alqadhi A.M., Abdullah C., AK A.S., Olaiya N., Abdat M. (2021). The Role of Biopolymer-Based Materials in Obstetrics and Gynecology Applications: A Review. Polymers.

[22] Fernandes S.C., Freire C.S., Silvestre A.J., Neto C.P., Gandini A., Berglund L.A., Salmén L. (2010). Transparent chitosan films reinforced with a high content of nanofibrillated cellulose. Carbohydr. Polym..

[23] Gupta P., Singh B., Agrawal A.K., Maji P.K. (2018). Low density and high strength nanofibrillated cellulose aerogel for thermal insulation application. Mater. Des..

[24] Majidi R., Taghiyari H., Abdolmaleki D. (2019). Molecular dynamics simulation evaluating the hydrophilicity of nanowollastonite on cellulose. J. Struct. Chem..

[25] Takeshita S., Yoda S. (2018). Upscaled preparation of trimethylsilylated chitosan aerogel. Ind. Eng. Chem. Res..

[26] Li Q., Renneckar S. (2011). Supramolecular structure characterization of molecularly thin cellulose I nanoparticles. Biomacromolecules.

[27] Khan R.A., Salmieri S., Dussault D., Uribe-Calderon J., Kamal M.R., Safrany A., Lacroix M. (2010). Production and properties of nanocellulose-reinforced methylcellulose-based biodegradable films. J. Agric. Food Chem..

[28] Wildan M.W., Lubis F.I. (2021). Fabrication and Characterization of Chitosan/Cellulose Nanocrystal/Glycerol Bio-Composite Films. Polymers.

[29] Li Q., Zhou J., Zhang L. (2009). Structure and properties of the nanocomposite films of chitosan reinforced with cellulose whiskers. J. Polym. Sci. Part B Polym. Phys..

[30] Zhang T., Zhang Y., Wang X., Liu S., Yao Y. (2018). Characterization of the nano-cellulose aerogel from mixing CNF and CNC with different ratio. Mater. Lett..

[31] Hajaligol M., Waymack B., Kellogg D. (2001). Low temperature formation of aromatic hydrocarbon from pyrolysis of cellulosic materials. Fuel.

[32] Pasangulapati V., Ramachandriya K.D., Kumar A., Wilkins M.R., Jones C.L., Huhnke R.L. (2012). Effects of cellulose, hemicellulose and lignin on thermochemical conversion characteristics of the selected biomass. Bioresour. Technol..

[33] Ko E., Kim H. (2020). Preparation of chitosan aerogel crosslinked in chemical and ionical ways by non-acid condition for wound dressing. Int. J. Biol. Macromol..

[34] Neto C.d.T., Giacometti J.A., Job A.E., Ferreira F.C., Fonseca J.L.C., Pereira M.R. (2005). Thermal analysis of chitosan based networks. Carbohydr. Polym..

[35] Fan X., Li Y., Li X., Wu Y., Tang K., Liu J., Zheng X., Wan G. (2020). Injectable antibacterial cellulose nanofiber/chitosan aerogel with rapid shape recovery for noncompressible hemorrhage. Int. J. Biol. Macromol..

[36] Sahariah P., Snorradóttir B.S., Hjálmarsdóttir M.Á., Sigurjónsson Ó.E., Másson M. (2016). Experimental design for determining quantitative structure activity relationship for antibacterial chitosan derivatives. J. Mater. Chem. B.

[37] Atiqah M., Gopakumar D.A., FAT O., Pottathara Y.B., Rizal S., Aprilia N., Hermawan D., Paridah M., Thomas S., Abdul Khalil H.P.S. (2019). Extraction of cellulose nanofibers via eco-friendly supercritical carbon dioxide treatment followed by mild acid hydrolysis and the fabrication of cellulose nanopapers. Polymers.

[38] Martins M., Barros A.A., Quraishi S., Gurikov P., Raman S., Smirnova I., Duarte A.R.C., Reis R.L. (2015). Preparation of macroporous alginate-based aerogels for biomedical applications. J. Supercrit. Fluids.

